# Zebrafish Model for Screening Antiatherosclerosis Drugs

**DOI:** 10.1155/2021/9995401

**Published:** 2021-06-22

**Authors:** Jichun Han, Rui Zhang, Xiaofeng Zhang, Jing Dong, Minghan Chen, Yumin Pan, Zixian Liao, Min Zhong, Jingwen He, Feiqiang Wang, Yunyun Yue, Jing Shang

**Affiliations:** School of Traditional Chinese Pharmacy, China Pharmaceutical University, Nanjing, 211198 Jiangsu, China

## Abstract

This study is aimed at establishing a zebrafish model of AS, which can be applied for high-throughput screening anti-AS drugs. A zebrafish AS model was induced by high cholesterol diet (HCD) and lipopolysaccharide (LPS). In the early stage of modeling, HCD induced zebrafish to show some early symptoms similar to human AS, mainly cholesterol accumulation, vascular inflammation, lipid metabolism disorder, and oxidative stress. In addition to lipid metabolism disorders, LPS also induced the same symptoms. And when HCD and LPS exist at the same time, these AS symptoms in zebrafish become more severe. When the modeling time reached 45 days, HCD and LPS induce the formation of plaques in zebrafish blood vessels, and these plaques contain fibrous tissue and lipids, which are similar to human AS plaques. We also evaluated the efficacy of some anti-AS drugs (atorvastatin, aspirin, and vitamin C) through these zebrafish AS models. The results found that atorvastatin can significantly reduce the symptoms of AS induced by HCD and LPS, and aspirin and vitamins can significantly reduce the symptoms of AS induced by LPS. It is feasible to use zebrafish to establish an AS model, and the zebrafish AS model can be used for high-throughput screening of anti-AS drugs.

## 1. Introduction

Atherosclerosis (AS) is believed to be one of the main causes of cardiovascular disease and death [1, 2]. The prominent feature of AS is that cholesterol gradually accumulates on the blood vessel wall to form plaque, which causes vascular stenosis and sclerosis [3]. The involving animal models more often use mouse, rat, rabbit, miniature pig, and nonhuman primates [4, 5]. Large animal models such as pigs and nonhuman primates are suitable for AS research because their vascular lesion morphology and lipid metabolism are similar to that in humans. The disadvantages of these models include long modeling times, high cost, complex experimental procedures, and difficulties in obtaining large amounts of data [5, 6]. Small mammalian animals such as rabbits and mice are cheaper to rear, and mice can be easily manipulated genetically. However, these small animal AS models can only provide endpoint results, and the long modeling time is unsuitable for large-scale screening of potential therapeutic drugs and targets [7, 8]. For large-scale anti-AS drug screening, the difficulty of achieving high-throughput screening is the common deficiency of all the above AS animal models. AS seriously endangers people's lives and health, while the types of drugs used to treat AS are obviously insufficient. Therefore, it is necessary to establish an animal model that is reliable, low-cost, and high-throughput screening of anti-AS drugs.

Zebrafish are a unique vertebrate model that has not only the characteristics of invertebrate models (small size, powerful genetic tractability, high fecundity, ease of maintenance, and relatively low cost) but also the advantage of evolutionary conservation in mammals. In addition, zebrafish larvae are optically transparent until about 30 days postfertilization (dpf), which enables observation of disease progression in vivo [9]. Thus, zebrafish are invaluable for studying the development and physiology of vertebrate as well as modeling human diseases. Our previous studies have shown that a zebrafish hypercholesterolemia model induced by a high-cholesterol diet can be applied to reflect the early symptoms of human AS that cholesterol accumulation in blood vessels can be observed in vivo [10]. However, there was no hallmark phenomenon of plaque appears. Therefore, this study is aimed at establishing the zebrafish AS model by a variety of factors.

To explore the potential of zebrafish for AS-related studies, we treated zebrafish with high cholesterol diet (HCD), lipopolysaccharide (LPS), and HCD+LPS, respectively. The results showed that HCD, LPS, and HCD+LPS can all induce AS in zebrafish, and all 3 zebrafish AS models can be applied to screen anti-AS drugs.

## 2. Materials and Methods

### 2.1. Test Compounds, Chemicals, and Reagents

Nile red and cholesterol were purchased from Sigma-Aldrich (St. Louis, MO, United States). Fluorescent cholesteryl (CholEsteryl BODIPY™ FL C12) was purchased from Thermo Fisher Scientific (Waltham, Massachusetts, U.S.). 2′,7′-Dichlorofluorescin diacetate was purchased from Beijing Solarbio Science & Technology Co., Ltd. (Beijing, China). Triglyceride (TG) kit, total cholesterol (TC) kit, superoxide dismutase (SOD) kit, and malondialdehyde (MDA) kit were purchased from Nanjing Jiancheng Biological Institute (Nanjing, China). Ethyl 3-aminobenzoate methanesulfonate was purchased from Sigma-Aldrich (St. Louis, MO, United States).

### 2.2. Experimental Animal

Adult zebrafish were maintained according to standard laboratory conditions. The following lines were used: AB, Tg(*fli1a*:EGFP), Tg(*lyz*:DsRED2), and Tg(*mpx*:EGFP). All zebrafish were immersed in 0.03% ethyl 3-aminobenzoate methanesulfonate solution for ≤3 min to induce anaesthesia. All zebrafish were euthanized by immersion in overdose of ethyl 3-aminobenzoate methanesulfonate (1.33 g/L). Animal work was approved by the local Animal Welfare and Ethical Review Body at China Pharmaceutical University.

### 2.3. Zebrafish AS Model

In the present study, HCD, LPS, and HCD+LPS were used to treat zebrafish to establish an AS model. The specific operations of the 3 zebrafish AS models are as follows:

1. HCD-induced AS model (HCD-AS): the 5 dpf zebrafish larvae were raised in egg water and fed with HCD containing 8% cholesterol for 45 days
2. LPS-induced AS model (LPS-AS): the 5 dpf zebrafish larvae were raised in egg water containing 10 *μ*g/mL LPS and fed with normal basic feed for 45 days
3. HCD+LPS-induced AS model (HCD+LPS-AS): the 5 dpf zebrafish larvae were raised in egg water containing 10 *μ*g/mL LPS and fed with HCD containing 8% cholesterol for 45 days

### 2.4. Detection of Plaques in Zebrafish Blood Vessels

After 45 days of modeling and 24 hours of fasting, the zebrafish were euthanized with an overdose of ethyl 3-aminobenzoate methanesulfonate (1.33 g/L) and completely fixed with paraformaldehyde. HE staining, EVG staining, and Oil red O staining were performed to detect plaque formation in the blood vessels of zebrafish.

### 2.5. Detection of Cholesterol Accumulation

Tg(*fli1a*:EGFP) zebrafish were used to build AS zebrafish models, and 10 *μ*g/g fluorescent cholesterol was added to the feed. Five days after the model was built, the zebrafish were immersed in 0.03% ethyl 3-aminobenzoate methanesulfonate solution for ≤3 min to induce anaesthesia. The cholesterol accumulation in blood vessels was observed under a fluorescence microscope.

### 2.6. Detection of Lipid Levels

Wild-type AB-line zebrafish were used to build AS zebrafish models. After 5 days of modeling and 24 hours of fasting, the zebrafish were immersed in 0.03% ethyl 3-aminobenzoate methanesulfonate solution for ≤3 min to induce anaesthesia. Nile red staining was performed to detect lipid levels.

Wild-type AB-line zebrafish were used to build AS zebrafish models. After 5 days of modeling and 24 hours of fasting, the zebrafish were euthanized with an overdose of ethyl 3-aminobenzoate methanesulfonate (1.33 g/L). Briefly, 5 larvae from each group were randomly selected and sacrificed as one sample, and six samples were prepared for testing each index. The levels of triglyceride (TG) and total cholesterol (TC) were measured using commercial assay kits following the manuscript's protocol (Jiancheng, Nanjing, China).

### 2.7. Detection of Inflammation

In the present study, the macrophage infiltration in the blood vessels, numbers of neutrophils, and the mRNA expression of VCAM-1b were determined to reflect the inflammation in zebrafish.

In brief, Tg(*lyz*:DsRED2) zebrafish were used to establish AS zebrafish models. Five days after the model was built, the zebrafish were immersed in 0.03% ethyl 3-aminobenzoate methanesulfonate solution for ≤3 min to induce anaesthesia. The macrophage infiltration in blood vessels was observed under a fluorescence microscope. Similarly, Tg(*mpx*:EGFP) zebrafish were used for the observation of the number of neutrophils.

Three AS models established by wild-type AB-line zebrafish were used to measure the mRNA expression of *vcam-1b*, *tnf-α*, and *il-6*. After 5 days of modeling and 24 hours of fasting, the zebrafish were euthanized with an overdose of ethyl 3-aminobenzoate methanesulfonate (1.33 g/L). Five larvae from each group were randomly selected and sacrificed as one sample, and six samples were prepared for testing each index. Total RNA of the sample was extracted using the TRIzol reagent (Invitrogen, Carlsbad, CA, USA) according to the manufacturer's instructions. The RNA was reverse transcribed using the PrimeScript RT Master Mix (Perfect Real Time) following the manufacturer's protocol. The resultant cDNA was applied as the template for quantitative PCR analyses in the Thermal Cycler Dice® Real Time System (Takara Bio Inc., Shiga, Japan) with the following sets of primers: primers for quantitative real-time PCR (qPCR) were designed by Primer3 software and shown in Supplementary Materials Table S1. The mRNA expression data are expressed as a relative expression ratio normalized to GAPDH.

### 2.8. Detection of Oxidative Stress

Wild-type AB-line zebrafish were used to build AS zebrafish models. Five days after the model was built, the zebrafish were immersed in 0.03% ethyl 3-aminobenzoate methanesulfonate solution for ≤3 min to induce anaesthesia. The ROS activity was detected by DCFH-DA.

After 5 days of modeling and 24 hours of fasting, the zebrafish were euthanized with an overdose of ethyl 3-aminobenzoate methanesulfonate (1.33 g/L). Five larvae from each group were randomly selected and sacrificed as one sample, and six samples were prepared for testing each index. The superoxide dismutase (SOD) activity and malondialdehyde (MDA) level were measured by commercial assay kits following the manuscript's protocol (Jiancheng, Nanjing, China).

### 2.9. Statistical Analysis

Data are presented as the mean ± standard deviation. Statistical differences were determined using analysis of variance (ANOVA), where *p* < 0.05 was considered statistically significant. All statistical analysis was performed by GraphPad Prism 8.0 software (San Diego, CA, USA).

## 3. Results

### 3.1. Early AS Symptoms in Zebrafish

**Figure 1:**
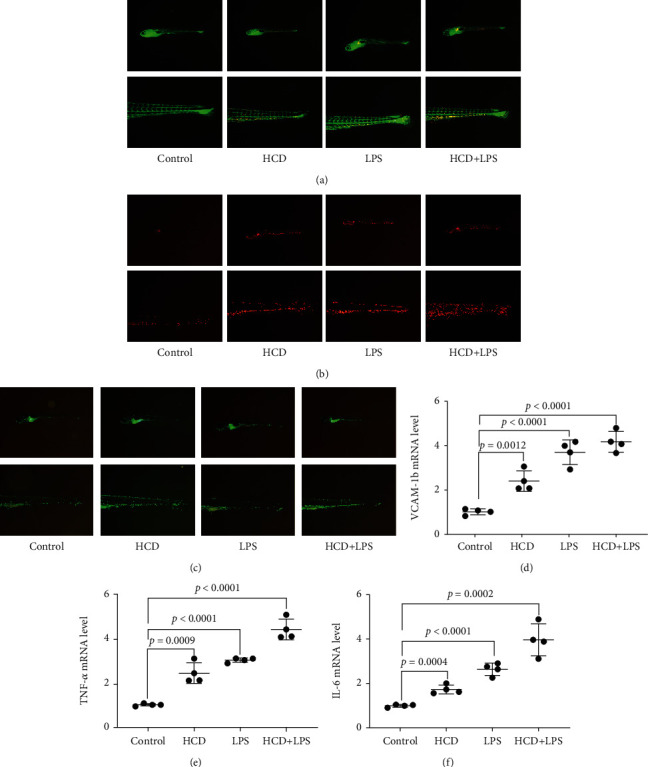
Early symptoms of AS zebrafish. (a) Cholesterol accumulates in the blood vessels of AS zebrafish. The numbers of macrophages (b) and neutrophils (c) in the blood vessels of AS zebrafish. The mRNA expressions of *vcam-1b* (d), *tnf-α* (e), and *il-6* (f) of 3 AS zebrafish. Representative images and bar graphs (mean ± SD) were expressed, *n* = 4.

Cholesterol accumulation and macrophage infiltration in blood vessels are the early symptoms of AS. Therefore, we used Tg(*fli1a*:EGFP) and Tg(*lyz*:DsRED2) zebrafish to establish an AS model to observe cholesterol accumulation and macrophage infiltration in blood vessels. As shown in Figure 1(a) and Figure 1(b), we found that both HCD and LPS induced cholesterol accumulation and macrophage infiltration in blood vessels, and when HCD and LPS exist at the same time, cholesterol accumulation and macrophage infiltration in blood vessels are more serious. AS is considered to be a chronic inflammatory disease, and early vascular inflammation is one of the risk factors for AS. Therefore, we used Tg(*mpx*:EGFP) zebrafish to establish an AS model to observe neutrophils in blood vessels to reflect inflammation. As shown in Figure 1(c), a large number of neutrophils appeared in the blood vessels of HCD-AS and LPS-AS zebrafish, and this phenomenon is more serious in HCD+LPS-AS zebrafish. qPCR was performed to measure the mRNA expressions of some vascular adhesion molecules and inflammatory factors in AS zebrafish. As depicted in Figures 1(d)–1(f), the mRNA expressions of *vcam-1b*, *tnf-α*, and *il-6* of these 3 AS zebrafish were significantly increased, and the HCD+LPS-AS zebrafish had the highest expression.

### 3.2. Disorders of Lipid Metabolism and Oxidative Stress in AS Zebrafish

**Figure 2:**
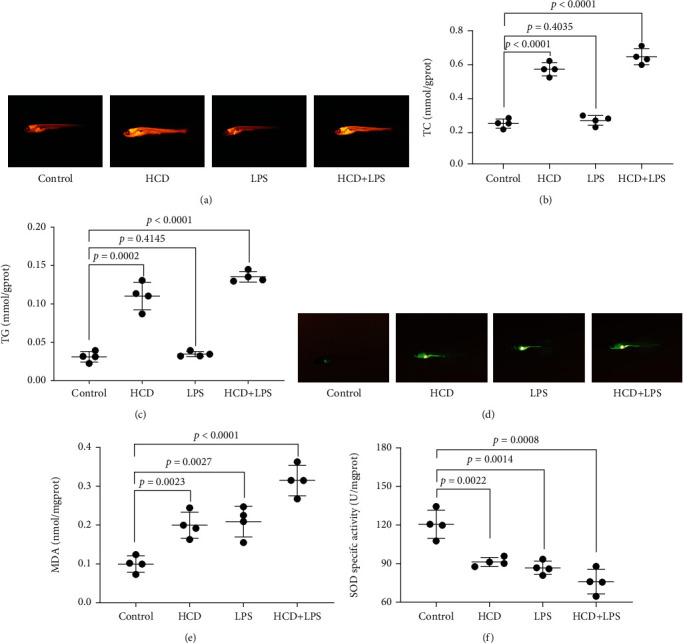
Disturbance of lipid metabolism and oxidative stress in early AS zebrafish. Effects of HCD, LPS, and HCD+LPS on the lipid level (a), TC (b), and TG (c) content of AS zebrafish. Effects of HCD, LPS, and HCD+LPS on the ROS activity (d), MDA content (e), and SOD activity (f) of AS zebrafish. Representative images and bar graphs (mean ± SD) were expressed, *n* = 4.

Hyperlipidemia is also one of the risk factors for AS. We used Nile red staining to observe the lipid levels of zebrafish. As shown in Figure 2(a), HCD significantly increased the lipid level of zebrafish, but LPS did not cause an abnormal increase in lipids. We also tested the content of TC and TG in zebrafish. As shown in Figures 2(b) and 2(c), HCD significantly increased the content of TC and TG, but LPS had no effect on the content of TC and TG. AS is also related to oxidative stress. In order to observe whether there is oxidative stress in AS zebrafish, we tested the ROS activity, MDA content, and SOD activity of each group of zebrafish. As shown in Figures 2(d)–2(f), the DCFH-DA probe detection of ROS activity shows that the ROS activity of these 3 AS zebrafish is effectively strengthened, and the HCD+LPS-AS zebrafish exhibited the highest ROS activity. The MDA level of these 3 AS zebrafish are significantly increased, and the HCD+LPS-AS zebrafish had the highest MDA level. The SOD activity of these 3 AS zebrafish is significantly weakened, and the HCD+LPS-AS zebrafish exhibited the lowest SOD activity.

### 3.3. Plaque Formation in AS Zebrafish

**Figure 3:**
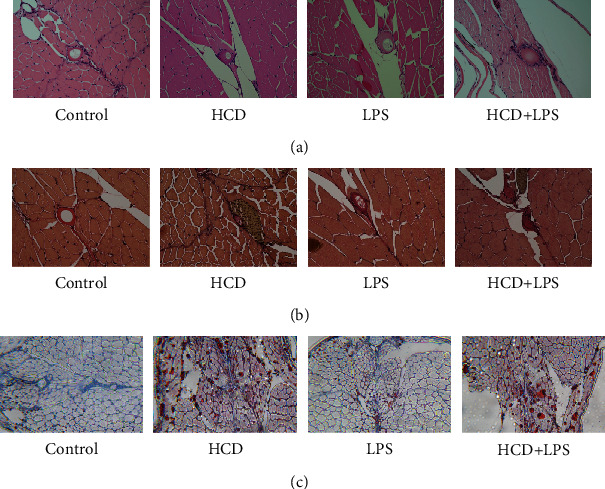
Plaque formation of AS zebrafish. (a) The results of HE staining showed that plaques were formed in the blood vessels of the 3 AS zebrafish, causing vascular stenosis. (b) The results of EVG staining showed that the plaques of the 3 AS zebrafish all contained fibrous tissue. (c) The results of Oil red O staining showed that the plaques of HCD-AS zebrafish and HCD+LPS-AS zebrafish contained a large amount of lipids, while the lipid components of LPS-AS zebrafish plaques were few. Representative images were expressed, *n* = 4.

The above research results show that the early symptoms of the zebrafish AS model are similar to those of humans. But the hallmark of AS is the formation of plaque in blood vessels. In order to observe whether plaques can form in AS zebrafish blood vessels, we extended the modeling time to 45 days and used HE staining, EVG staining, and Oil red O staining to detect plaques in zebrafish blood vessels. The results of HE staining showed that HCD and LPS induced plaques to form in zebrafish blood vessels, causing vascular stenosis, and the vascular stenosis caused by HCD+LPS was more serious (Figure 3(a)). The results of EVG staining also showed that there were plaques in the blood vessels of 3 AS zebrafish, and the plaques contained fibrous tissue (Figure 3(b)). The Oil red O staining results showed that the plaques in the blood vessels of HCD-AS zebrafish and HCD+LPS-AS zebrafish contained a large amount of lipids, but LPS-AS zebrafish did not (Figure 3(c)).

The above results indicate that the pathogenesis and symptoms of zebrafish AS are similar to human AS.

### 3.4. Efficacy of Positive Drugs on Early Symptoms of AS Zebrafish

**Figure 4:**
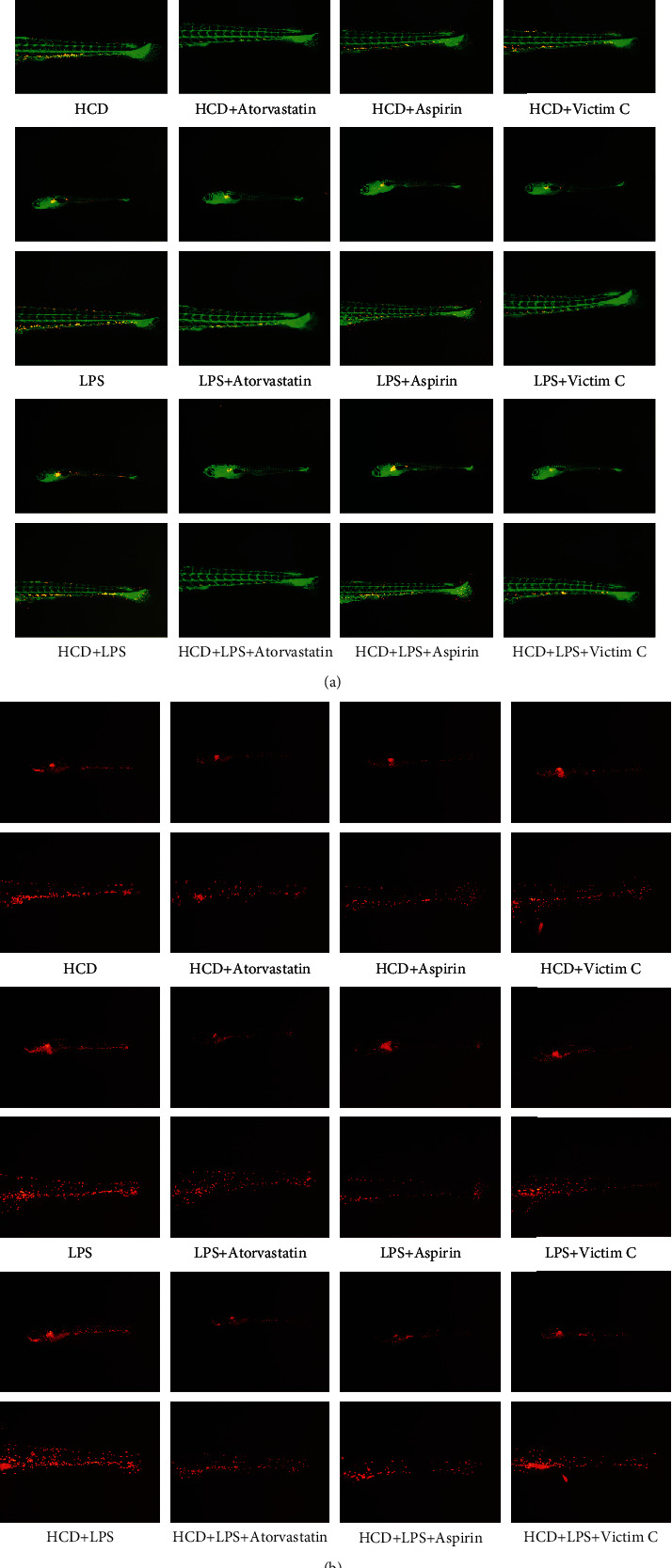
Effects of atorvastatin, aspirin, and vitamin C on early symptoms of AS zebrafish. (a) Effects of atorvastatin, aspirin, and vitamin C on the cholesterol accumulation. (b) Effects of atorvastatin, aspirin, and vitamin C on the numbers of macrophages. Representative images were expressed, *n* = 4.

As depicted in Figure 4(a), in HCD-AS zebrafish, atorvastatin, aspirin, and vitamin C reduced the accumulation of cholesterol in blood vessels, and atorvastatin had the best effect. In LPS-AS zebrafish, atorvastatin, aspirin, and vitamin C reduced the accumulation of cholesterol in blood vessels, and the efficacy of the three drugs is similar. In HCD+LPS-AS zebrafish, atorvastatin, aspirin, and vitamin C reduced the accumulation of cholesterol in blood vessels, and atorvastatin had the best effect. We also observed that the 3 drugs can effectively reduce the number of macrophages in the blood vessels of 3 AS zebrafish (Figure 4(b)).

### 3.5. Efficacy of Positive Drugs on Inflammatory Response, Lipid Metabolism Disorder, and Oxidative Stress in Early AS Zebrafish

**Figure 5:**
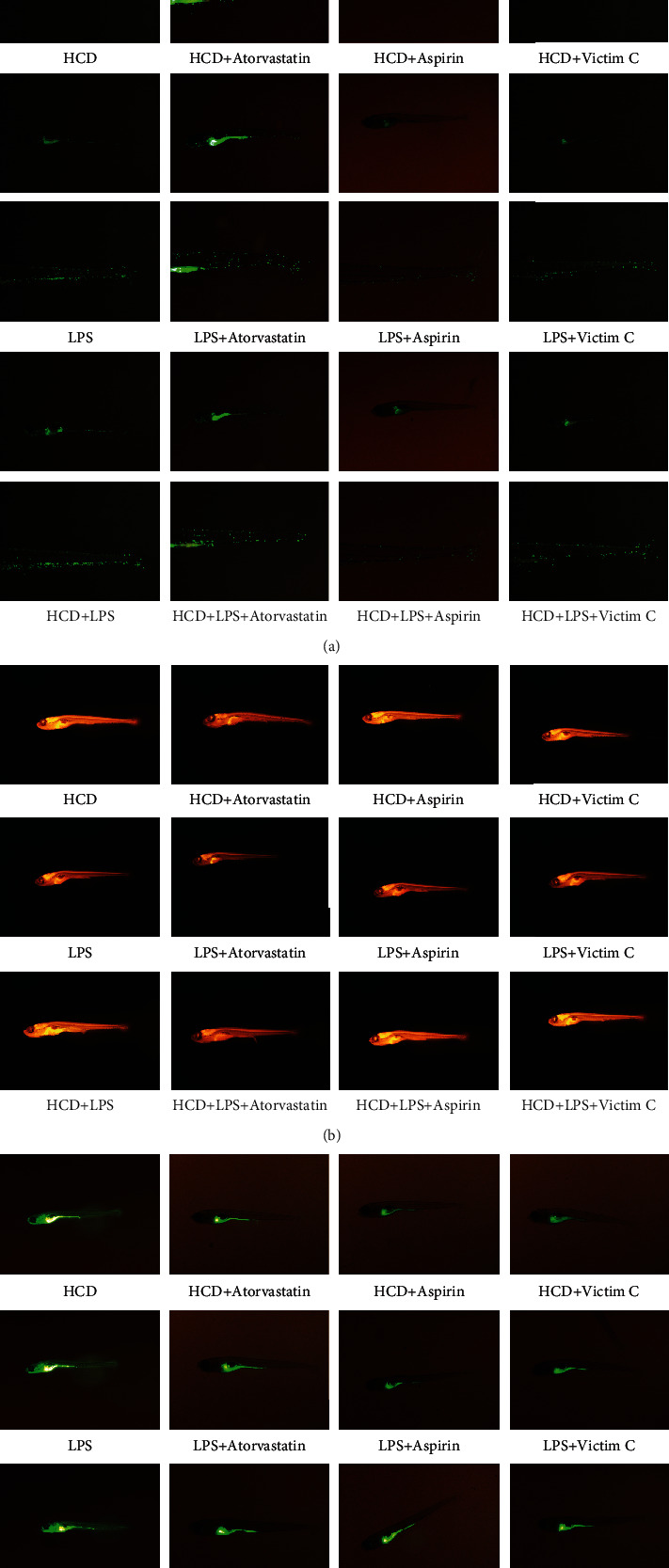
Effects of atorvastatin, aspirin, and vitamin C on inflammation, lipid metabolism, and oxidative stress in early AS zebrafish. (a) Effects of atorvastatin, aspirin, and vitamin C on the numbers of neutrophils. (b) Effects of atorvastatin, aspirin, and vitamin C on the lipid level. (c) Effects of atorvastatin, aspirin, and vitamin C on the ROS activity. Representative images were expressed, *n* = 4.

Three positive drugs improved the inflammatory response of 3 AS zebrafish. As depicted in Figure 5(a), atorvastatin, aspirin, and vitamin C significantly reduced the number of neutrophils in the blood vessels of 3 AS zebrafish, and aspirin is the most effective. We also used qPCR to detect the effects of these drugs on inflammatory factors (*vcam-1b*, *tnf-α*, and *il-6)* in several AS zebrafish. The results showed that atorvastatin, aspirin, and vitamin C all significantly reduced the mRNA expression of *vcam-1b*, *tnf-α*, and *il-6* in 3 AS zebrafish (Supplementary Materials Figure S1).

Atorvastatin improved lipid metabolism disorders in HCD-AS zebrafish and HCD+LPS-AS zebrafish. As shown in Figure 5(b), both HCD-AS zebrafish and HCD+LPS-AS zebrafish have abnormally increased lipid levels. Atorvastatin effectively reduced the lipid levels of HCD-AS zebrafish and HCD+LPS-AS zebrafish, but aspirin and vitamin C are not effective in reducing lipid levels. In addition, the same results were obtained by testing the content of TG and TC in AS zebrafish (Supplementary Materials Figure S2).

Three positive drugs improved the oxidative stress of 3 AS zebrafish. As shown in Figure 5(c), atorvastatin, aspirin, and vitamin C significantly reduced the ROS activity of 3 AS zebrafish. We also tested the MDA content and SOD activity. As shown in Supplementary Materials Figure S3, atorvastatin, aspirin, and vitamin C significantly reduced the MDA content of the three AS zebrafish and increased the SOD activity.

### 3.6. Effect of Positive Drugs on AS Zebrafish Plaque Formation

**Figure 6:**
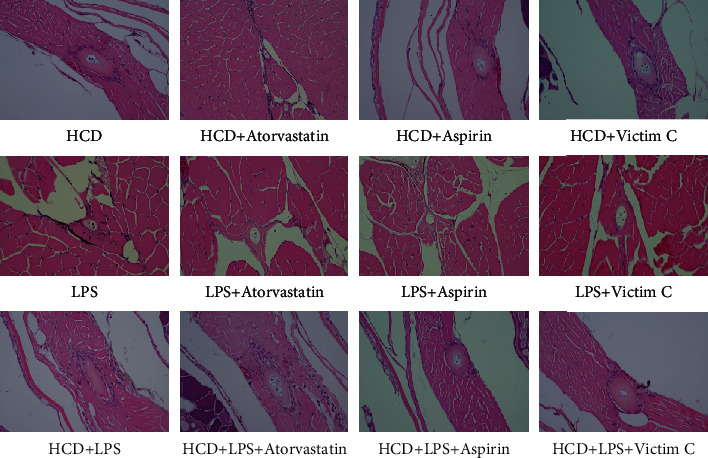
HE staining to detect the effects of atorvastatin, aspirin, and vitamin C on AS zebrafish plaque formation. Representative images were expressed, *n* = 4.

As shown in Figure 6, HE staining results show that atorvastatin effectively reduces plaque formation in the blood vessels of HCD-AS zebrafish, but aspirin and vitamin C have no effect on plaque formation in the blood vessels of HCD-AS zebrafish. Atorvastatin, aspirin, and vitamin C significantly reduced plaque formation in the blood vessels of LPS-AS zebrafish, and the efficacy of the three drugs is similar. Atorvastatin effectively reduces plaque formation in the blood vessels of HCD+LPS-AS zebrafish, but aspirin and vitamin C can only slightly improve the plaque formation in the blood vessels of HCD+LPS-AS zebrafish.

**Figure 7:**
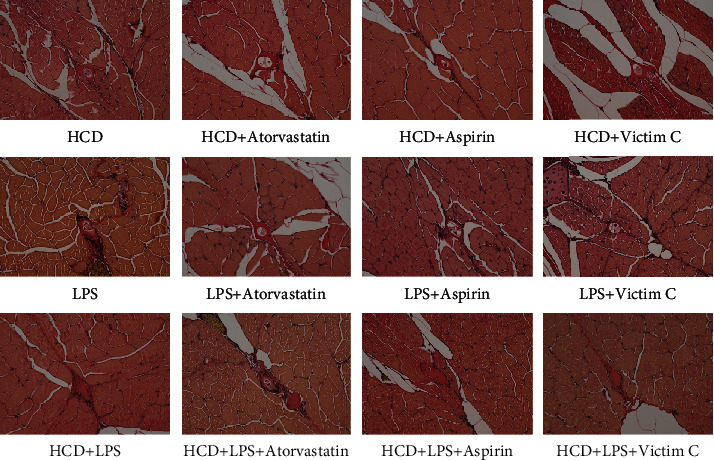
EVG staining to detect the effects of atorvastatin, aspirin, and vitamin C on fibrous tissue in AS zebrafish plaques. Representative images were expressed, *n* = 4.

As shown in Figure 7, atorvastatin effectively reduced the fibrous tissue in the HCD-AS zebrafish plaques and improved vascular stenosis, but aspirin and vitamin C can only slightly reduce the fibrous tissue in the HCD-AS zebrafish plaque. Atorvastatin, aspirin, and vitamin C reduced the fibrous tissue in HCD-AS zebrafish plaques and improve blood vessel stenosis, and aspirin had the best effect. In HCD+LPS-AS zebrafish, only atorvastatin can slightly reduce the fibrous tissue in zebrafish vascular plaques, while aspirin and vitamin C are almost ineffective.

**Figure 8:**
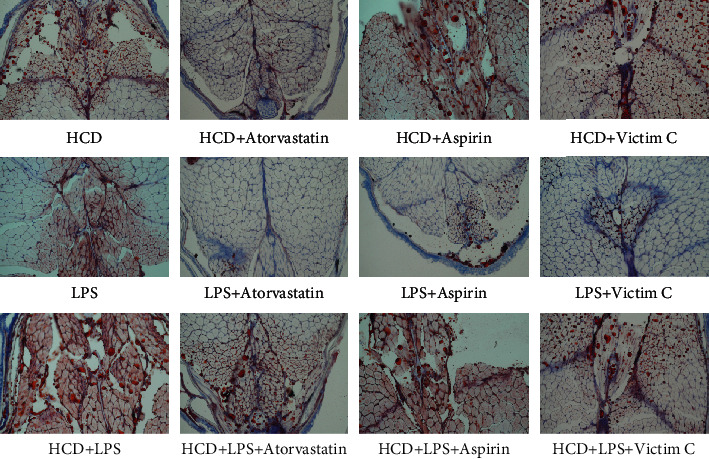
Oil red O staining to detect the effects of atorvastatin, aspirin, and vitamin C on lipids in AS zebrafish plaques. Representative images were expressed, *n* = 4.

The Oil red O staining results are shown in Figure 8, and atorvastatin significantly reduces the lipid accumulation in the blood vessels of the three AS zebrafish, but aspirin and vitamin C have little effect.

## 4. Discussion

AS is a progressive inflammatory disease characterized by the accumulation of lipids (mainly cholesterol) in the arterial vessel wall, eventually forming plaques and narrowing the vessel lumen [11, 12]. Hypercholesterolemia is an important hazard factor for AS [13]. Excessively high cholesterol in the blood can not only directly accumulate on the blood vessel wall but also damage the blood vessels and induce inflammation to further aggravate the accumulation of cholesterol and eventually give rise to the formation of plaque [14]. HCD is currently a common way to establish AS. After HCD treatment for 3 months, plaques will appear in the blood vessels of experimental animals [15, 16]. As a small vertebrate, zebrafish has a high degree of similarity with human genes by 87%. This model organism showed outstanding advantages and has been extensively applied in human disease research [9]. Studies have found that zebrafish can be used for researches in the early AS [17]. After feeding zebrafish HCD for 10 days, there will be cholesterol accumulation in the blood vessels similar to those in patients with early AS, but no plaques were observed [10, 16]. In the present study, we fed zebrafish HCD to establish an HCD-induced AS (HCD-AS) model. In the early stages (about 5 days after feeding), cholesterol accumulation appeared in the blood vessels of the HCD-AS zebrafish. At the same time, we also observed a large number of macrophages in the blood vessel species. These symptoms are consistent with the early symptoms of human AS. The results of the zebrafish in vivo examination showed that with the extension of modeling time, the accumulation of cholesterol in the blood vessels gradually aggravated, and the pathological section results showed that the blood vessel intima thickened and eventually developed into plaques. Compared with human AS plaques, we found that HCD-AS zebrafish plaques contain a lot of lipids and fibers, which are similar to human AS plaques. A significant increase was also observed in lipid levels in HCD-AS zebrafish, which is consistent with most AS patients.

AS is a chronic inflammatory disease of the vascular wall, and many immune cells are involved in its occurrence and development, such as macrophages and neutrophils [18, 19]. Macrophages play an important role in the whole process of AS, while in different stages, the role is completely different. In the early stage of AS, the infiltration of macrophages into blood vessels directly form foam-like cells and eventually form plaques. Besides, macrophages can also induce inflammation via releasing some inflammatory factors (such as *vcam-1b*, *tnf-α*, and *il-6*) to promote the formation of plaques [20]. In the late stages of AS, macrophage apoptosis contributes to the plaque rupture and thrombosis and induces cardiovascular diseases such as cerebral and myocardial infarction [20, 21]. In the present study, we observed a large number of macrophages accumulated in the blood vessels of HCD-AS zebrafish. In addition, the mRNA expression of *vcam-1b*, *tnf-α*, and *il-6* of HCD-AS zebrafish increased significantly. All these indicate that the changes of HCD-AS zebrafish macrophages are similar to those of human AS.

Neutrophil adhesion is considered an essential process for the early response of acute vascular inflammation [19]. Endothelial cell dysfunction, the initiation of AS, would be the immediate consequence of neutrophil adhesion to endothelial cells [22]. Neutrophils release some inflammatory factors such as TNF-*α*, IL-1, and IL-8 [23]. These inflammatory factors not only result in inflammatory damage to blood vessels but also strengthen the neutrophil adhesion to endothelial cells further induced by vascular cell adhesion molecules. Furthermore, they also directly activate neutrophils. Neutrophil activation will produce a large amount of ROS, which directly causes oxidative damage to blood vessels and promotes the formation of plaques [24]. In the present study, masses of neutrophils were found in the blood vessels of HCD-AS zebrafish, and the mRNA expression of inflammatory factors (*v tnf-α* and *il-6*) and vascular cell adhesion molecule (*vcam-1b*) upregulated significantly. HCD-AS zebrafish also exhibited oxidative stress, as manifested by the significant increase of ROS and MDA content and the significant decrease of SOD activity. All these illustrated that the changes above of HCD-AS zebrafish neutrophils are similar to those of human AS neutrophils.

In addition to hypercholesterolemia, numerous factors could cause the occurrence of AS, such as bacterial infections [25, 26]. By detecting bacteria in AS plaques, bacteria were found to induce AS by releasing LPS [27, 28]. In the present study, zebrafish were treated with LPS to establish an LPS-AS model. The results showed that LPS can also induce cholesterol accumulation, and plaques similar to AS patients formed in blood vessels over time. In accordance with HCD-AS zebrafish, masses of macrophages and neutrophils appeared in the blood vessels of LPS-AS zebrafish, and the mRNA expression of inflammatory factors and vascular cell adhesion molecules was also appreciably upregulated. In the meantime, LPS-AS zebrafish experienced oxidative stress. But no significant difference in the lipid level of LPS-AS zebrafish was measured. Interestingly, when treated with both HCD and LPS at the same time, the AS symptoms of zebrafish are particularly more serious than those of HCD-AS and LPS-AS zebrafish. These results showed that the usage of LPS or HCD+LPS in zebrafish can simulate a human-like AS model.

At present, the main clinical drugs for the treatment of AS are statins [29, 30]. In this study, we used atorvastatin, a type of statin, to pretreat HCD-AS, LPS-AS, and HCD+LPS-AS zebrafish. Consequently, atorvastatin appreciably reduced the plaque formation of these 3 AS zebrafish, and this effect was best presented in HCD-AS zebrafish. We have also observed that atorvastatin improves other symptoms of these 3 AS zebrafish, such as reducing lipid metabolism disorders, macrophage infiltration, neutrophil recruitment, inflammation, and oxidative stress.

Researches have suggested that antiplatelet drug has a good preventive effect on AS [31, 32]. We used an antiplatelet drug called aspirin to pretreat HCD-AS, LPS-AS, and HCD+LPS-AS zebrafish. The results showed that aspirin significantly reduced plaque formation in LPS-AS zebrafish and improved LPS-induced cholesterol accumulation, macrophage infiltration, neutrophil recruitment, inflammation, and oxidative stress. Although aspirin significantly improved the oxidative stress and inflammation in HCD-AS and HCD+LPS-AS zebrafish, aspirin showed no effective effect on reducing the plaque formation and cholesterol accumulation. These data illustrated that aspirin can improve some symptoms of AS zebrafish but cannot reduce the formation of plaques, which is similar to the actual situation of aspirin treatment of the clinical AS.

It is common knowledge that vitamin C possesses the effect of protecting the structure and function of blood vessel walls, which is beneficial to the prevention and treatment of cardiovascular diseases [33]. Therefore, this study used vitamin C to pretreat HCD-AS, LPS-AS, and HCD+LPS-AS zebrafish. The results showed that vitamin C significantly reduced plaque formation in LPS-AS zebrafish and improved LPS-induced cholesterol accumulation, macrophage infiltration, neutrophil recruitment, inflammation, and oxidative stress. Although vitamin C significantly improves the oxidative stress and inflammation in HCD-AS zebrafish and HCD+LPS-AS zebrafish, aspirin cannot effectively reduce the plaque formation and cholesterol accumulation in the blood vessels of HCD-AS zebrafish and HCD+LPS-AS zebrafish. These results indicate that vitamin C can improve some symptoms of AS but cannot reduce the formation of plaques, which is similar to the actual situation of vitamin C treatment of AS.

## 5. Conclusions

In summary, zebrafish is suitable for simulating AS models, and the AS zebrafish models could be used for screening anti-AS drugs and related basic research.

## Data Availability

All data used to support the findings of this study are included within the article.

## Supplementary Materials

Supplementary Materials Table S1: primer sequence. Figure S1: effects of atorvastatin, aspirin, and vitamin C on the mRNA expression of *vcam-1b* (*A-C*), *tnf-α* (*D-F*), and *il-6* (*G-I*) in 3 AS zebrafish. Figure S2: effects of atorvastatin, aspirin, the TC (*A-C*), and TG (*D-F*) content of 3 AS zebrafish. Figure S3: effects of atorvastatin, aspirin, and vitamin C on the MDA content (*A-C*), and SOD activity (*D-F*) of 3 AS zebrafish. Representative images and bar graphs (mean ± SD) are expressed.
