# Supplementary material for: Zebrafish Model for Screening Antiatherosclerosis Drugs
Source: Oxid Med Cell Longev. 2021 Jun 22;2021:9995401. doi: 10.1155/2021/9995401 (PMC8245221; doi:10.1155/2021/9995401)
Supplement: Supplementary Materials — Table S1: primer sequence. Figure S1: effects of atorvastatin, aspirin, and vitamin C on the mRNA expression of vcam-1b (A-C), tnf-α (D-F), and il-6 (G-I) in 3 AS zebrafish. Figure S2: effects of atorvastatin, aspirin, the TC (A-C), and TG (D-F) content of 3 AS zebrafish. Figure S3: effects of atorvastatin, aspirin, and vitamin C on the MDA content (A-C), and SOD activity (D-F) of 3 AS zebrafish. Representative images and bar graphs (mean ± SD) are expressed. [file 9995401.f1.zip › Fig S2.docx]

**Fig S2A**

**Fig S2B**

**Fig S2C**

**Fig S2D**

**Fig S2E**

**Fig S2F**
