# Supplementary material for: Zebrafish Model for Screening Antiatherosclerosis Drugs
Source: Oxid Med Cell Longev. 2021 Jun 22;2021:9995401. doi: 10.1155/2021/9995401 (PMC8245221; doi:10.1155/2021/9995401)
Supplement: Supplementary Materials — Table S1: primer sequence. Figure S1: effects of atorvastatin, aspirin, and vitamin C on the mRNA expression of vcam-1b (A-C), tnf-α (D-F), and il-6 (G-I) in 3 AS zebrafish. Figure S2: effects of atorvastatin, aspirin, the TC (A-C), and TG (D-F) content of 3 AS zebrafish. Figure S3: effects of atorvastatin, aspirin, and vitamin C on the MDA content (A-C), and SOD activity (D-F) of 3 AS zebrafish. Representative images and bar graphs (mean ± SD) are expressed. [file 9995401.f1.zip › Supplementary Materials (1).docx]

**Zebrafish Model for Screening Anti-atherosclerosis Drugs**

Jichun Han ^1*^, Rui Zhang ^1*^, Xiaofeng Zhang ^1^, Jing Dong ^1*^, Minghan Chen ^1^, Yumin Pan ^1^, Zixian Liao ^1^, Min Zhong ^1^, Jingwen He ^1^, Feiqiang Wang ^1^, Yunyun Yue ^1#^, Jing Shang ^1#^

*^1^ School of Traditional Chinese Pharmacy, China Pharmaceutical University, Nanjing 211198, Jiangsu, China*

^*^ Jichun Han and Rui Zhang have contributed equally to this article.

^#^ Correspondence: Jing Shang (E-mail: [shangjing21cn@cpu.edu.cn](mailto:shangjing21cn@cpu.edu.cn)),

Yunyun Yue (E-mail: [xgwkqt@126.com](mailto:xgwkqt@126.com))

**Table S1** Primer sequence

| **Gene** | **Species** |  | **Primer sequence (5’ → 3’)** |
| --- | --- | --- | --- |
| *vcam-1b* | Danio rerio | Forward | ACCTTCGGTTACGCTGGATG |
|  |  | Reverse | CGTATTCCTGGGAGGTGCTTT |
| *tnf-α* | Danio rerio | Forward | GGTGTGGATCAGGCATTCCA |
|  |  | Reverse | TCTCACTGCATCGGCTTTGT |
| *il-6* | Danio rerio | Forward | ATGACGGCATTTGAAGGGGT |
|  |  | Reverse | TCAGGACGCTGTAGATTCGC |
| *gapdh* | Danio rerio | Forward | GGGTGATGCAGGTGCTACTT |
|  |  | Reverse | GGCAGGTTTCTCAAGACGGA |


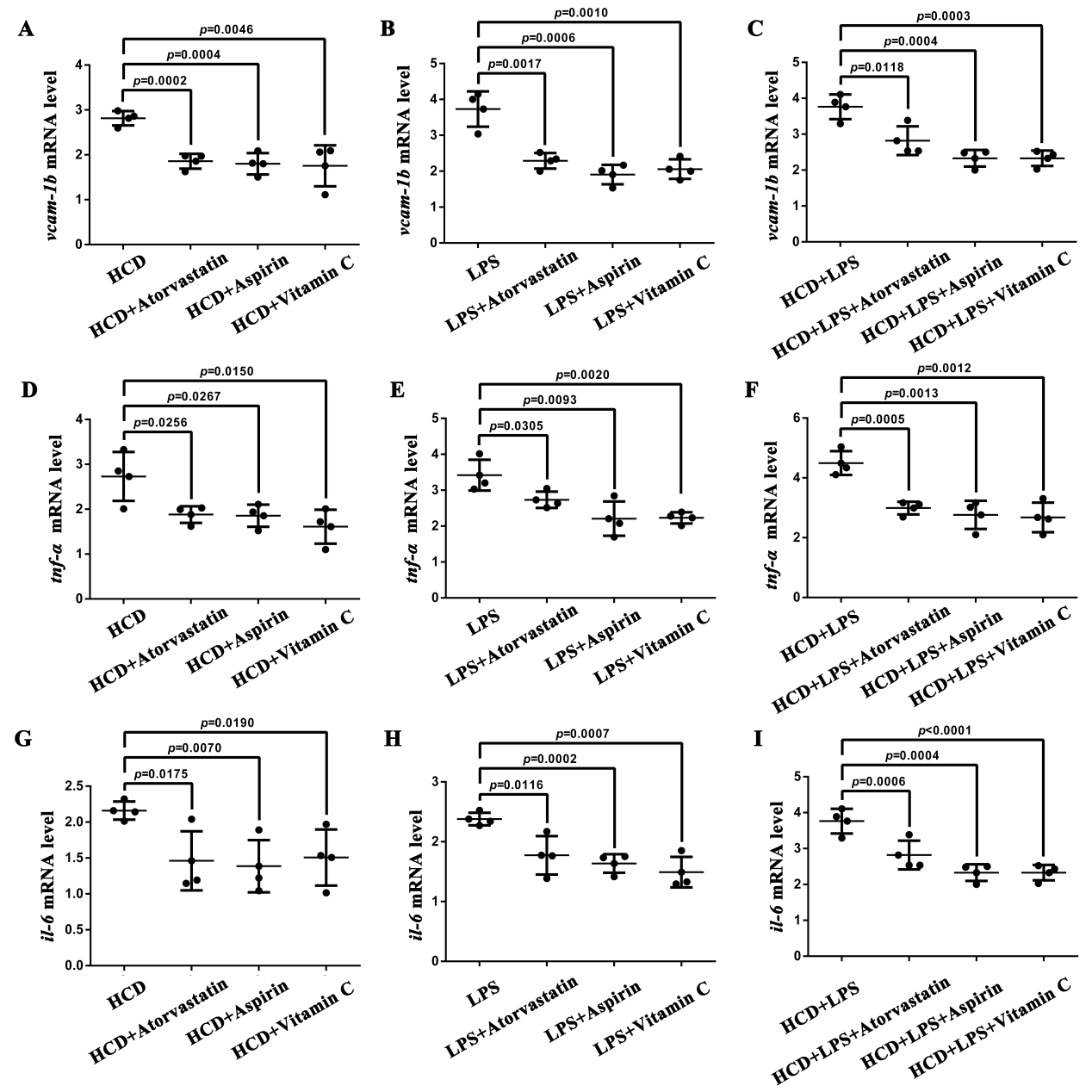


**Figure S1**  Effects of atorvastatin, aspirin, and vitamin C on the mRNA expression of *vcam-1b* (*A-C*), *tnf-α* (*D-F*), and *il-6* (*G-I*) in 3 AS zebrafish. *n*=4.


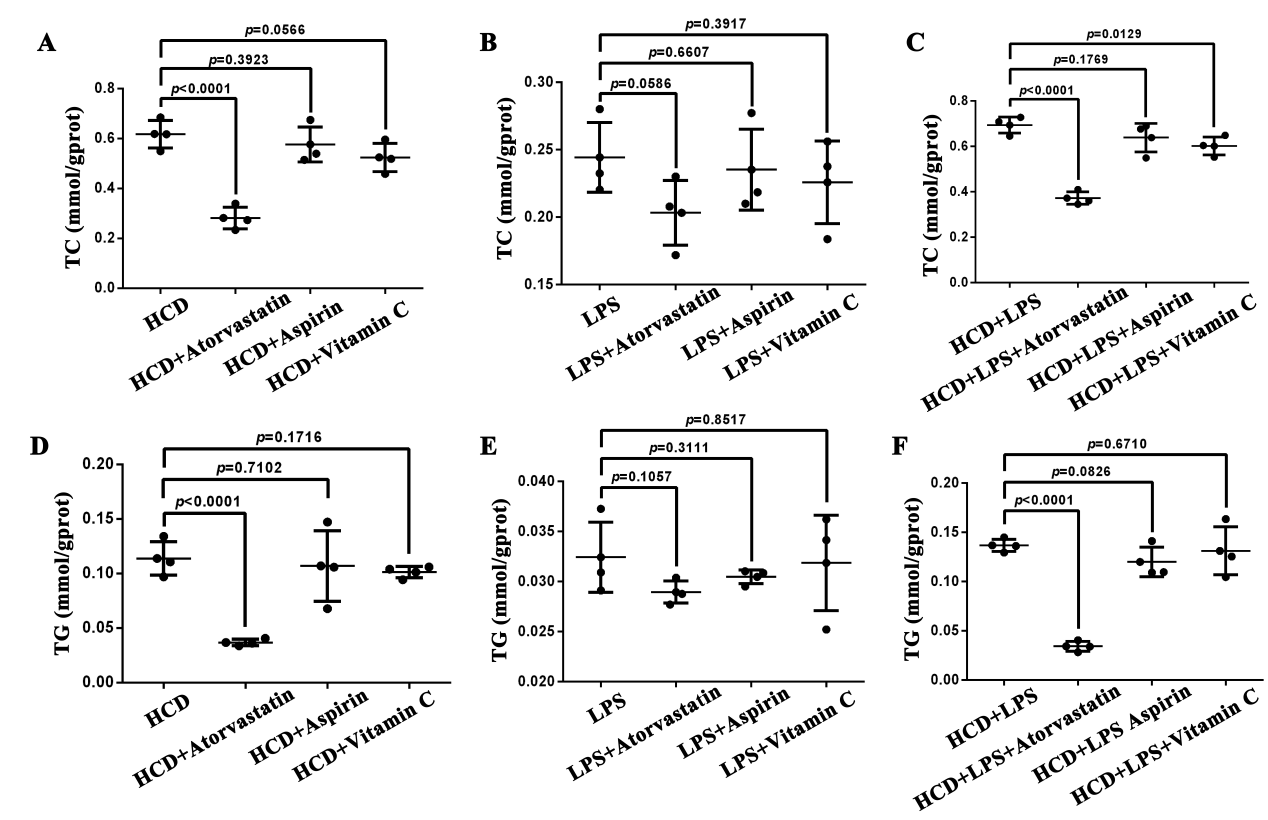


**Figure S2** Effects of atorvastatin, aspirin, and vitamin C on the TC (*A-C*), and TG (*D-F*) content of 3 AS zebrafish. *n* = 4.


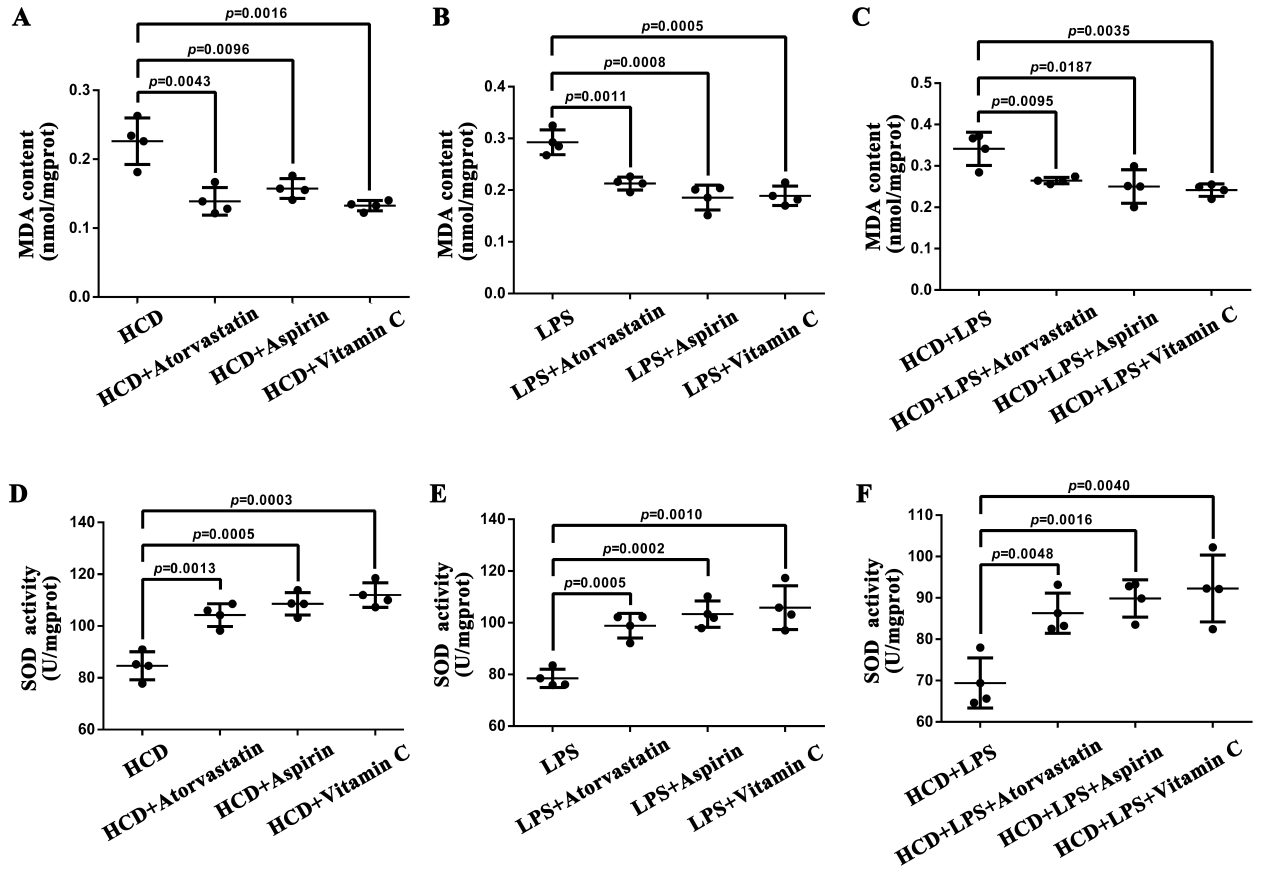


**Figure S3**  Effects of atorvastatin, aspirin, and vitamin C on the MDA content (*A-C*), and SOD activity (*D-F*) of 3 AS zebrafish. Representative images and bar graphs (mean ± SD) are expressed, *n* = 4.
